# Age-Based Comparison of Head and Neck Cancer Characteristics and Reconstructive Outcomes: Retrospective Review of 286 Patients

**DOI:** 10.3390/medicina62050822

**Published:** 2026-04-25

**Authors:** Hyun Il Kang, Seok Joon Lee, Feras AlShomer, Tae Suk Oh, Jong Woo Choi, Woo Shik Jeong

**Affiliations:** Department of Plastic and Reconstructive Surgery, Asan Medical Center, University of Ulsan College of Medicine, Seoul 05505, Republic of Korea; soreekang@naver.com (H.I.K.); sjlee_ps@naver.com (S.J.L.); dr.fshomer@gmail.com (F.A.); tasuko@amc.seoul.kr (T.S.O.); pschoi@amc.seoul.kr (J.W.C.)

**Keywords:** head and neck cancer, reconstruction, age stratification, functional outcome, retrospective review

## Abstract

*Background and Objectives*: Head and neck cancer (HNC) frequently necessitates reconstructive surgery due to defects following oncologic resection. The influence of age on reconstructive outcomes in head and neck cancer remains controversial. This study aimed to evaluate the impact of age on oncologic characteristics, reconstructive strategies, and functional outcomes following microvascular free flap reconstruction. *Materials and Methods*: A retrospective review was conducted on 286 patients who underwent free flap reconstruction for head and neck cancer between 2016 and 2020. Patients were stratified into three age groups: <40 years, 40–60 years, and >60 years. Demographic characteristics, tumor features, reconstructive approaches, complications, and functional outcomes—including postoperative dietary tolerance and tube feeding dependency—were analyzed. *Results*: The oral cavity was the most common tumor site across all age groups. Advanced-stage tumors (T4) were more frequently observed in older patients (>60 years), although the difference was not statistically significant (*p* = 0.0575). The overall flap survival rate was 98.6%. The mean hospital stay was 24.6 ± 15.86 days and was significantly longer in the >60-years group (*p* < 0.001). Postoperative dietary tolerance was comparable across age groups, with 56.8% of patients resuming a regular diet. Tube feeding dependency was slightly higher in the >60-years group but did not reach statistical significance (*p* = 0.1599). *Conclusions*: Age alone does not significantly affect reconstructive outcomes following microvascular free flap reconstruction for head and neck cancer. Despite a higher prevalence of comorbidities in and longer hospital stays for older patients, flap success rates and functional outcomes were comparable across age groups.

## 1. Introduction

Head and neck cancer (HNC) is one of the most common cancer types with a mortality rate of approximately 6.5 per 100,000 people reported in 2020 [1]. Most tumors are squamous cell carcinomas (SCCs) originating from the oral cavity [2]. Demographic evaluation identified several risk factors that were shown to have strong association with the development of such tumors, among which were alcohol consumption, tobacco use, and seropositivity with human papillomavirus [2]. Surgical resection remains the mainstay of treatment for head and neck cancer [3,4,5]. The complexity of these lesions with the potential involvement of various subsites in the vicinity often makes surgical defects after ablation quite complex, with a potential effect on the overall patient functional and esthetic outcomes [6,7]. Modern advancements in reconstructive microsurgery have revolutionized the management of patients with HNC and allowed for a tailored reconstructive approach that ensures proper functional recovery with a success rate ranging between 90% and 98% [8,9].

Studies have investigated the incidence of HNC among different age groups and showed that it has a peak incidence around the fifth and sixth decades of life. Moreover, approximately 25% of patients with an advanced age (>70 years) were more likely to be affected by cancer [10,11]. Age as a confounding variable has been investigated in previous reports for its effect on tumor behavior and treatment outcomes; however, there remains no internationally agreeable distinction between young and old age groups [12,13,14,15]. It was reported that female patients younger than 45 years were more likely to develop oral tongue SCC than male ones of the same age, regardless of other associated risk factors [16,17]. However, among those between 50 and 60 years old, male patients were more likely to develop buccal mucosa carcinoma [17,18]. Additionally, when reviewing the overall survival and tumor characteristics between different age groups, heterogenous reports showed neither a worse nor better prognosis in young patients than older ones and a lack of effect in between [19,20,21]. However, in a meta-analysis, Tagliabue M. et al. showed that young patients (<45 years of age) with tongue carcinoma were associated with better overall survival but with a higher chance of local recurrence than older patients without adjusting for over-confounding variables, such as TNM Category and treatment modality [18]. This clearly shows a site-specific and tumor-specific effect that warrants further investigation.

Regarding the reconstructive approach related to patients with HNC, age was also investigated in several reports that concluded that advanced age lacks a significant effect with a safety profile similar to that of young age groups. However, with advancing age, the need for meticulous preoperative counseling and adjustment of comorbid conditions was shown to be of great value in maintaining such success [22,23,24]. Moreover, most reports discussed only the feasibility of flap reconstruction with a complication profile and lacked a clear comparison in terms of flap-related outcomes, treatment modality effect, and overall functional outcomes between different age groups. Therefore, in this study, we analyzed and presented our reconstructive outcomes in managing various HNC defects and compared them between young age (<40 years), mid-age (40–60 years), and advanced age (>60 years) groups.

## 2. Materials and Methods

A retrospective chart review of all patients with HNC who underwent microvascular reconstruction between 2016 and 2020 was done. This study was approved by the institutional review board of Asan Medical Center (#2022-0534, 22 April 2022) and performed in accordance with the principles of the Declaration of Helsinki. Informed consent was waived because of the retrospective nature of the study and the use of de-identified clinical data. The patients were stratified into three groups according to age: <40, 40–60, and >60-years groups. The patients’ demographics, including age and sex, past medical history, and smoking history; diagnosis with cancer site and pathology; and cancer characteristics, including pathological TNM classification (based on the AJCC 8th edition), cancer recurrence, features of post-ablative defects, the reconstructive approach, complications, hospital stay, and the overall functional outcomes classified according to diet regimen, were included and analyzed. Recorded comorbidities included diabetes mellitus, hypertension, cardiovascular disease, and other major systemic conditions when available in the institutional database. Functional dietary outcome was assessed at the latest available follow-up visit and categorized as regular diet, soft diet, liquid diet, or PEG-tube dependence. Patients with incomplete medical records and those lost to follow-up were excluded. We excluded patients with pre-existing neurological conditions which could influence the surgical outcome from the study. Patients who underwent reconstruction for secondary defects related to osteonecrosis or for salvage treatment of previously recurrent head and neck cancer at the time of index surgery were also excluded. However, tumor recurrence occurring during postoperative follow-up after the index reconstruction was recorded and analyzed as an oncologic outcome.

Data were collected into an Excel sheet, and descriptive analysis was used for qualitative variables, which are expressed as percentages, and quantitative variables, which are expressed as means and standard deviations. The chi-square test or Fisher’s exact test was used for comparing qualitative variables, and analysis of variance was used for quantitative variables. All statistical analyses were performed using Statistical Package for the Social Sciences, version 27.0 (IBM Corp. Armonk, NY, USA). *p*-values of less than 0.05 were used to denote statistical significance.

## 3. Results

In this study, 286 patients with a mean age of 59.46 ± 12.83 years were included. Of the 286 patients, 204 (71.3%) were males. Comorbid conditions were present in 144 (50.3%) patients, of which diabetes mellitus was observed in 47 (16.4%) patients and hypertension in 97 (33.9%) patients. Furthermore, 142 (49.7%) patients had a history of smoking. The patients were then stratified into three groups based on their age: young age (<40 years), mid-age (40–60 years), and advanced age (>60 years) groups.

The median follow-up duration for the entire cohort was 37 months. The median follow-up durations in the <40-years, 40–60-years, and >60-years groups were 40.5, 32.5, and 25.5 months, respectively.

In the young age group (<40 years), 24 (8.39%) patients with a mean age of 30.79 ± 6.07 years with an equal male-to-female ratio (50%) were included. No patients had a history of any comorbid conditions; however, six (25%) patients had a history of smoking upon this assessment. In the mid-age (40–60 years) group, 118 (41.25%) patients with a mean age of 53.22 ± 4.79 years were included, with most patients (*n* = 83, 70.3%) being males. Comorbid conditions in this age group included hypertension, which was present in 26 (22.0%) patients, and diabetes mellitus, which was present in 13 (11%) patients. Moreover, 58 (49.2%) patients had a history of smoking. In the advanced age group (>60 years), 144 (50.34%) patients with a mean age of 69.35 ± 5.91 years were included, with most patients being male (*n* = 109, 75.7%). A review of the comorbid conditions in this age group showed that hypertension was present in 71 (49.3%) patients, diabetes mellitus was present in 34 (23.6%) patients, and 78 (54.2%) patients had a history of smoking. Comparing the three groups, a statistically significant difference in the prevalence of hypertension, diabetes mellitus, and smoking history was observed between the three groups, with *p*-values of <0.001, =0.0018, and =0.0299, respectively (Table 1).

The primary tumor site was also analyzed, and the oral cavity was the most common location, presented in 178 (62.24%) patients. This was followed by the oropharynx in 43 (15.03%) patients, the hypopharynx in 36 (12.59%) patients, and the larynx in 21 (7.34%) patients. When analyzing the different tumor locations in relation to age, the hypopharynx and larynx were the most common cancer sites in the advanced age group (>60 years) compared with the other groups with a statistically significant difference (*p* = 0.0245). Additionally, the oral cavity was the most common cancer site in the young and mid-age groups compared with that in the advanced age group. The data are summarized in Table 2. The T and N Categories presented in Table 2 refer to the pathological classification based on the final surgical specimen.

When reviewing the tumor characteristics, most lesions (*n* = 129, 45.1%) were moderately differentiated SCC, followed by well-differentiated SCC in 65 (29.7%) patients and poorly differentiated SCC in 27 (9.4%) patients, among others. It was noticed that most poorly differentiated lesions were present in the mid-age and advanced age groups. However, analysis of the tumor characteristics showed no significant difference between different age groups (*p* = 0.0575). When analyzing the tumor stage, the T4 Category was the most common tumor stage present in 103 (36.7%) patients, of whom most were in the advanced age group. One patient (0.4%) had a pathological T0 Category tumor, which reflects a complete pathological response following neoadjuvant treatment; reconstruction was performed based on the pre-treatment clinical staging and the resulting surgical defect.

Regarding the N Category, most patients had an N0 Category tumor (*n* = 151, 53.6%). Further analysis of the different age groups in relation to both the T and N Categories showed no statistically significant difference, with *p*-values of 0.2528 and 0.4429, respectively. Tumor recurrence was also reviewed, and among the patients under study, 58 (20.3%) had recurrence in their follow-up period, of whom 31 were in the advanced age group. However, no significant difference was observed between the three age groups with a *p*-value of 0.6314 (Table 2).

We further analyzed the reconstructive approach used in managing past cancer in the analyzed population and found that the most common flap used was the radial forearm free flap (RFFF) in 126 (44.1%) patients, followed by the anterolateral thigh (ALT) flap in 109 (38.1%) patients and the fibula osteocutaneous flap in 15 (5.2%) patients, among other options (Table 3). Of the 286 patients under study, only 19 (6.6%) patients developed early flap compromise that required immediate revision with complete salvage, of whom most were in the advanced age group; however, the difference was insignificant (*p* = 0.2912) when compared with the other age groups. Furthermore, the total flap success rate was approximately 98.6% with four flap losses (1.4%); no significant difference was observed between the age groups under study (*p* = 0.7815). Other complications present were hematoma in seven (2.5%) patients and wound dehiscence in 28 (9.8%) patients, of whom most were in the advanced age group; however, the difference was insignificant when compared with the other age groups (*p* = 0.0564). We looked at the overall hospital stay, and we found that the population under study had a mean hospital stay of 24.6 ± 15.86 days. Further analysis showed a longer hospital stay in the advanced age (>60 years) group with 26.6 ± 18.66 days than in the young age group with 21.46 ± 8.4 days and in the mid-age group with 22.81 ± 12.79 days; the difference was statistically significant (*p* < 0.001). The data are summarized in Table 3.

**Figure 1:**
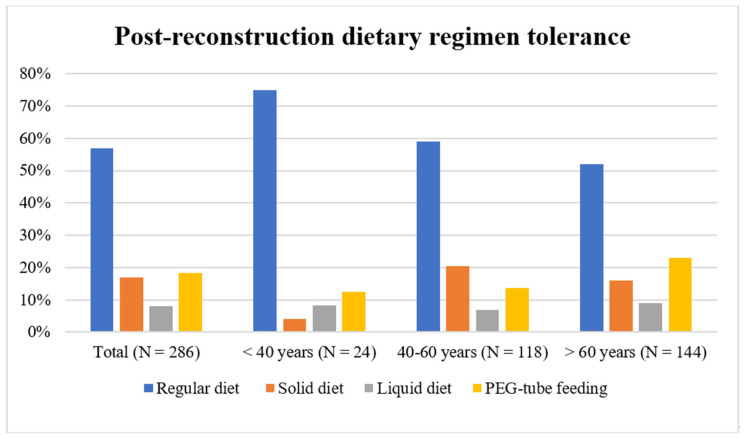
Summary of the overall post-reconstruction dietary regimen tolerance outcomes in the different age groups. Abbreviation: PEG, percutaneous endoscopic gastrostomy.

We further analyzed the functional outcomes related to swallowing and found that 162 (56.84%) patients could resume intake of a regular diet, whereas 48 (16.84%) patients could tolerate a soft diet only and 23 (8.07%) patients could consume a liquid diet only, and finally, 52 (18.25%) patients were percutaneous endoscopic gastrostomy tube-dependent, most of whom were in the advanced age group (n = 33). However, the difference between the different age groups was statistically insignificant (*p* = 0.1599). The results are summarized in Figure 1.

## 4. Discussion

The current series of age-stratified patients with HNC highlights the different tumor behaviors and the reconstructive outcomes across different age groups. We showed the different HNC characteristics and management outcomes with in-depth analysis of three age groups. It is important to mention that the definitions of age limit in relation to HNC are quite heterogeneous in the literature and no agreeable cutoff point has been observed [12,13,14,15]. Some studies have shown their outcomes with the use of an arbitrary number that ranges from 20 or 30 years to 45 years for the young age group and more than that for the old age group [12,13,14,15]. The ambiguity of making such a distinction between young and old makes it difficult to draw precise conclusions, and we believe that further analysis of patients age-stratified into more than two groups is of great value to understand such an effect, which was presented in this series.

The different HNC risk factors, such as smoking and alcohol consumption, between different age groups could help establish appropriate tumor surveillance and early detection, particularly in the young age group [25]. Additionally, some reports have pointed out an increasing trend of HNC occurrence in young patients, even without known risk factors. This was shown to have a significant association with young female patients [26]. In this study, smoking history was evaluated as a potential risk factor. The prevalence of smoking was highest in the advanced age group, whereas only six patients (25.0%) in the young age group had a history of smoking. This difference was statistically significant among the age groups, and smoking history was more common in male patients. However, detailed risk analysis that involved other possible factors was unfeasible due to the retrospective and heterogenous nature of the data; therefore, this warrants further investigation.

Regarding the cancer prognosis and histopathological characteristics, in one meta-analysis, it was pointed out that young patients with oral tongue cancer have an overall better prognosis but a significantly higher chance of cancer recurrence than old patients [18]. However, a recent systematic review on the effects of age on oral cancer prognosis showed heterogenous conclusions; in part, it supported the poor outcomes of oral cancer in the young age group for the case series reviewed, and simultaneously, it included a matched-pair analysis that concluded no significant difference between the different age groups analyzed [27]. Troeltzsch et al. presented their experience with patients with oral cavity cancer across different age groups in which cancer histopathological features were analyzed and showed that most patients with T4 cancer were in the 40–80-years age group. Moreover, most patients had an N0 disease with prevalent grade II tumor across all age groups; however, the difference was insignificant [20].

Although advanced disease constituted a large proportion of the cohort, a subset of patients underwent reconstruction for relatively limited primary tumors, including T1 lesions. This likely reflects the functional and anatomic importance of head and neck defects, in which even smaller tumors may require reconstruction depending on the site and extent of ablative surgery.

In our cohort, the oral cavity was the most common tumor site, particularly in the <40-years and 40–60-years groups, whereas hypopharyngeal and laryngeal tumors were more frequent in the advanced age group. In addition, T4 disease was more common in older patients, while N0 disease remained the most frequent nodal category overall, which is broadly consistent with previous reports [20]. Recurrence rates were 25.0% in the <40-years group, 17.8% in the 40–60-years group, and 21.5% in the >60-years group, without a statistically significant difference among groups. These findings are in keeping with previous studies showing that the prognostic effect of age in head and neck cancer remains heterogeneous and often becomes less clear after accounting for tumor-related factors [18,20,27]. Further case-matched analyses may help clarify this relationship. However, a subsite-specific analysis of reconstructive outcomes was not performed in the present study because the sample sizes within several tumor subsites were insufficient for reliable statistical comparison across age groups. Future studies with larger, site-specific cohorts are warranted to evaluate the potential influence of primary tumor location on reconstructive and functional outcomes.

Regarding the reconstructive approach, all patients in this series received free flap reconstruction with most patients receiving RFFF (126 (44.1%)), followed by ALT flaps in 109 (38.1%) patients. The flap success rate reached approximately 98.6% without a significant difference between the analyzed age groups in terms of flap loss or any other complications, indicating that age has no clear effect on such outcomes. This is in line with the results of previous studies that showed a similar finding with a safety profile similar to that of the young age group. Furthermore, the flap options used in the reconstructive approach were also similar to the options used in this series, as shown in one recent meta-analysis [23]. One important factor to consider is the need for proper preoperative optimization of the patients’ comorbid conditions to further improve the overall outcomes, leading to the concept of the biological age rather than the chronological age in terms of flap success and complication profile [22]. In our patient population, most comorbid conditions and smoking history occurred in the advanced age group with a significant difference compared with the other age groups. However, multivariate analysis of those factors in relation to the complication profile showed no significant association.

Hospital stay was among the factors analyzed, and we found that the overall mean hospital stay duration was approximately 24.6 ± 15.86 days, with the advanced age group having a significantly prolonged hospital stay with a mean duration of 26.6 ± 18.66 days. This was of interest as previous reports have shown mixed outcomes linking the complication profile to hospital stay, intensive care unit stay, or even general anesthesia duration [28]. Additionally, a meta-analysis by Ustun et al. could not calculate the mean duration of hospital stay from previous reports and could not find any association of this factor with any complications. However, the reported duration ranged from 18.7 to 22 days for the old age group compared with 3.6 to 24.4 days for the young age group [23]. This clearly shows the need for a controlled case-matched analysis that addresses the many confounding variables that might implicate the association of these durations with potential complications. The longer hospital stay observed in older patients may reflect a higher burden of comorbidities, slower postoperative recovery, a greater need for perioperative medical optimization, and more cautious discharge planning. Because flap-related complication rates were not significantly different across age groups, the prolonged hospitalization in older patients likely reflects differences in overall perioperative recovery rather than flap failure itself.

Although many studies have assessed the effect of age on the HNC profile along with surgical safety and complication profile, few have evaluated the effect of age on the functional outcomes. One study investigated the effect of advanced age (>70 years) on the timing of tracheostomy decannulation and resumption of oral feeding but failed to show any significant difference compared with the young age group [29]. Parsemain et al. showed similar findings in relation to tracheostomy decannulation but less frequent gastric tube removal in the older age (>70 years) group, but there were no statistically significant difference between the age groups analyzed [30]. Our analysis in relation to swallowing and enteral feeding dependency showed that 52 (18.25%) patients were gastric tube-dependent, most of whom were in the advanced age group, but with no significant difference compared with the other age groups, which is similar to the findings of previous reports [29,30].

This study has several limitations. First, its retrospective design is subject to inherent selection bias and limits the ability to fully control for confounding variables affecting HNC reconstruction outcomes. However, the data were derived from an electronically maintained database that included all patients who underwent HNC free flap reconstruction at our institution during the study period. Second, adjuvant therapy was not uniformly incorporated into the present analysis, although it may influence postoperative swallowing and nutritional outcomes. Third, the number of patients in the young age group was relatively small, whereas most patients belonged to the advanced age group, reflecting the epidemiology of HNC. Lastly, postoperative dietary tolerance may have been influenced by tumor site, extent of resection, and adjuvant treatment, which could not be fully adjusted for in the present retrospective analysis.

## 5. Conclusions

Considering the findings of this series and the literature, HNC across different age groups has a similar profile, regardless of the effect of age. In this cohort, age was not associated with inferior reconstructive outcomes after microvascular free flap reconstruction for head and neck cancer. Although older patients had a higher burden of comorbidities and longer hospital stays, flap survival and postoperative functional outcomes were comparable across age groups. Differences in oncologic characteristics were observed among age groups, but no clear age-related disadvantage in reconstructive outcome was demonstrated. However, meticulous preoperative screening and the optimization of comorbid conditions help improve such outcomes and decrease the potential prolonged hospital stay for those with advanced age. When considering the effect of age on the reconstructive outcomes, not only flap success and postoperative complications but also functional outcomes in terms of tube feeding dependency, tracheostomy, and speech along with quality of life should be considered as valuable endpoints in future studies.

## Figures and Tables

**Table 1:** Summary of the patients’ demographics and comorbid conditions.

	Total *n* = 286	<40 Years *n* = 24	40–60 Years *n* = 118	>60 Years *n* = 144	*p*-Value
**Age**	59.46 ± 12.83	30.79 ± 6.07	53.22 ± 4.79	69.35 ± 5.91	<0.001
**Sex**					0.0344
Female	82 (28.7)	12 (50)	35 (29.7)	35 (24.3)	
Male	204 (71.3)	12 (50)	83 (70.3)	109 (75.7)	
**Diabetes**	47 (16.4)	0 (0)	13 (11.0)	34 (23.6)	0.0018
**Hypertension**	97 (33.9)	0 (0)	26 (22.0)	71 (49.3)	<0.001
**Smoking**	142 (49.7)	6 (25.0)	58 (49.2)	78 (54.2)	0.0299

**Table 2:** Summary of the cancer-related characteristics and histopathological features.

	Total *n* = 286	<40 Years *n* = 24	40–60 Years *n* = 118	>60 Years *n* = 144	*p*-Value
**Cancer site**					<0.001
Oral cavity	178 (62.24)	18 (75)	84 (71.19)	76 (52.78)	
Oropharynx	43 (15.03)	3 (12.5)	17 (14.41)	23 (15.97)	
Hypopharynx	36 (12.59)	0 (0)	10 (8.47)	26 (18.06)	
Larynx	8 (2.8)	0 (0)	2 (1.69)	6 (4.17)	
Other locations *	21 (7.34)	3 (12.5)	5 (4.24)	13 (9.03)	
**Pathology**					0.0575
SCC, WD	65 (29.7)	6 (25)	40 (33.9)	39 (27.1)	
SCC, MD	129 (45.1)	11 (45.8)	41 (34.8)	77 (53.5)	
SCC, PD	27 (9.4)	1 (4.2)	14 (11.9)	12 (8.3)	
Others	45 (15.7)	6 (25)	23 (19.5)	16 (11.1)	
**T Category**					0.2528
0	1 (0.4)	0 (0)	0 (0)	1 (0.7)	
1	33 (11.7)	1 (4.4)	16 (13.9)	16 (11.2)	
2	57 (20.3)	3 (13)	28 (24.4)	26 (18.2)	
3	87 (31)	11 (47.8)	37 (32.2)	39 (27.3)	
4	103 (36.7)	8 (34.8)	34 (29.6)	61 (42.7)	
**N Category**					0.4429
0	151 (53.6)	12 (52.2)	65 (56)	74 (51.8)	
1	34 (12.1)	1 (4.4)	15 (12.9)	18 (12.6)	
2	65 (23.1)	7 (30.4)	28 (24.1)	30 (21)	
3	32 (11.4)	3 (13)	8 (6.9)	21 (14.7)	
**Recurrence**	58 (20.3)	6 (25)	21 (17.8)	31 (21.5)	0.6314

Abbreviations: SCC, squamous cell carcinoma; WD, well-differentiated; MD, moderately differentiated; PD, poorly differentiated. * Other locations include Nasopharynx, Nasal cavity, Maxillary sinus, Parotid gland.

**Table 3:** Summary of the defect reconstructive options used along with the overall flap survival and complication profile.

	Total N = 286	<40 Years N = 24	40–60 Years N = 118	>60 Years N = 144	*p*-Value
**Flap Option**					0.6176
RFFF	126 (44.1)	12 (50)	48 (40.7)	66 (45.8)	
ALT flap	109 (38.1)	10 (41.7)	45 (38.1)	54 (37.5)	
Fibula flap	15 (5.2)	0 (0)	4 (3.4)	11 (7.5)	
Gracilis flap	14 (4.9)	2 (8.3)	7 (5.9)	5 (3.5)	
SCIP flap	2 (0.7)	0 (0)	1 (0.9)	1 (0.7)	
VRAM flap	1 (0.4)	0 (0)	1 (0.9)	0 (0)	
Chimeric flap *	19 (6.7)	0 (0)	12 (10.7)	7 (4.9)	
**Flap Complications**					0.0575
Hematoma	7 (2.5)	1 (4.2)	1 (0.9)	5 (3.5)	0.2611
Dehiscence	28 (9.8)	2 (8.3)	6 (5.1)	20 (13.9)	0.0564
Flap loss	4 (1.4)	0 (0)	2 (1.7)	2 (1.4)	0.7815
**Flap Revision**	19 (6.6)	0 (0)	7 (5.9)	12 (8.3)	0.2912
**Hospital Stay (Days)**	24.6 ± 15.86	21.46 ± 8.4	22.81 ± 12.79	26.6 ± 18.66	<0.001

Abbreviations: RFFF, radial forearm free flap; ALT, anterolateral thigh flap; SCIP, superficial circumflex iliac artery perforator; VRAM, vertical rectus abdominis myocutaneous.

## Data Availability

The data supporting the findings of this study are available from the corresponding author upon reasonable request.
